# A novel method for bone fatigue monitoring and prediction

**DOI:** 10.1016/j.bonr.2019.100221

**Published:** 2019-08-17

**Authors:** Michelle L. Cler, Joseph J. Kuehl, Carolyn Skurla, David Chelidze

**Affiliations:** a Department of Mechanical Engineering, Baylor University, Waco, TX 76798, United States of America; b Department of Mechanical Engineering, University of Delaware, Newark, DE 19716, United States of America; c Department of Mechanical, Industrial and Systems Engineering, University of Rhode Island, Kingston, RI 02881, United States of America

**Keywords:** Bone health monitoring, Bone fatigue, Stress fractures, Dynamical systems, 00-01, 99-00

## Abstract

Bone fatigue, often manifest as stress fractures, is a common injury that plagues many individuals, adversely affect quality of life, and is an obstacle to extended human spaceflight. This manuscript details a pilot study that was conducted to determine if the Phase Space Warping (PSW) methodology could be used to monitor/predict fatigue failure in bone tissue. A Moon's beam experimental apparatus was used to perform variable amplitude fatigue tests on bovine bone specimens. Scanning electron microscopy was used to evaluate the fracture surface and identify the fracture type. The PSW method allowed for successful identification of the various damage modes and may lead to the development of a viable tool for predicting the health and fatigue life of bone.

## Highlights

• The Phase Space Warping method is applied to study the fatigue behavior of bone.
• A novel testing apparatus is utilized to chaotically fatigue bone under loading conditions which span both ductile and brittle fracture mechanics.
• Active damage mechanisms are identified by fracture classification and SEM imaging.
• Phase Space Warping is found to show great promising in tracking fatigue in bone when mixed damage modes are present.

## 1 Introduction

Stress fracture and other fatigue related bone phenomena are common injuries that plague many individuals. Stress fractures are small cracks in the bone that are caused by bone fatigue, and can lead to complete fracture, if left untreated. Stress fractures occur as a result of the bone remodeling process, which involves osteoclasts resorbing bone tissue and osteoblasts building new bone tissue, being unable to keep up with the micro-damage caused by the repetitive loading of the bone. Stress fractures may also result from loading bone tissue that has weakened during aging or prolonged exposure to extreme environments (for example space flight), due to an imbalance in the resorption and deposition rates of the bone remodeling process. Eventually, when the natural toughening mechanisms in the bone are unable to withstand the continued loading, the bone fails. Stress fractures occur most commonly in the femur, tibia, metatarsal and tarsal bones (Patel et al., Jan. 2011, Sanderlin and Raspa, 2003).

Three major factors that influence the prevalence of stress factors are:

1. Age/Health: As humans and other mammals age, the elastic modulus, toughness, and strength of mature bone tend to decrease. In humans, between the ages of 20 and 90 years, these properties decrease by approximately 2% every ten years (Sanderlin and Raspa, 2003). Thus, bone fractures are a concern in the elderly population, particularly in osteoporotic, post-menopausal women. Osteoporosis can occur in men and women; however, it generally progresses more rapidly in postmenopausal women. This condition is due to an increase in bone resorption and decrease in the deposition of bone during the remodeling process, which causes the bone to progressively become weaker and the fracture risk to increase (Sanderlin and Raspa, 2003). Remodeling continually occurs in bone tissue in order to keep the bone strong. Its main purpose is to repair bone and to adapt the tissue to applied mechanical loads. In healthy bone tissue, resorption and deposition are coupled and are controlled by hormones and proteins that are secreted by bone and bone marrow cells (Hadjidakis and Androulakis, 2006). However, as people age, hormone production in the body and other factors, such as physical activity level, change. These changes can cause more bone to be broken down than to be built up.
2. Intensive Training: Approximately 21% of American military recruits have been diagnosed with stress fractures, and approximately 21% of competitive runners were reported as acquiring stress fractures over the period of one year (Verheyen et al., Dec. 2006, Bennell et al., Mar. 1996). This injury not only occurs in runners and military recruits, but also plagues gymnasts, dancers, figure skaters, astronauts, the elderly, and animals such as racing horses and greyhound dogs.
3. Extreme Environments: Astronauts who spend a prolonged period of time in a microgravity environment are at an increased risk of bone fracture upon returning to an environment with gravity. This is due to disuse osteoporosis, which is similar to what postmenopausal osteoporotic individuals experience, but the bone loss in space is approximately 10 times greater and is due to a significantly decreased level of mechanical loading on the tissue (Ulbrich et al., 2014). At the end of a 4–6 month long space flight, the percentage of total bone loss is about 0–3 % with the largest percentage of bone loss in the weight bearing bones at 0–20 % (Ulbrich et al., Jul. 2014, Sambandam et al., May 2016, Iwamoto et al., 2005, Lang et al., Aug. 2006). Lang et al. performed a study on astronauts after 4–6 months in space and found that 1 year after the space flight, the bending and compressive strength of the bone were still approximately 10% less than the preflight strength (Lang et al., 2006).

Currently, stress fractures can only be diagnosed after they are large enough to be detected using radiography, magnetic resonance imaging, or triple-phase bone scintigraphy (Patel et al., 2011). Furthermore, to treat this injury, the patient wears a brace to restrict movement and modifies their activity level. This treatment allows the bone to remodel and heal where the fractures have occurred; however, it takes approximately 12 weeks for the injury site to fully heal and for the individual to return to his or her previous activity level (Patel et al., 2011).

In this manuscript, we report the results of a pilot study in which we endeavor to develop better methodologies for the monitoring of bone health and to predict bone failure caused by fatigue. Specifically, the Phase Space Warping (PSW) methodology is tested in an effort to determine if the damage state of the bone can be detected prior to failure, in the context of vibration analysis. If the damage state of bone can be monitored, then protocols for the mitigation or prevention of stress fractures may be developed. The intent of this pilot study was to test the PSW methodology, for the first time, on bone. Thus, as a prelude to in vivo and more human relevant efforts, the simplest of biologically relevant material was chosen for initial study; dehydrated bovine bone.

It should be noted that mechanical characterization of bone by vibration analysis is not a new idea. The Mechanical Response Tissue Analysis (MRTA) is a vibration analysis technique for measuring bending stiffness in the ulna and tibia that was developed at Stanford with support from NASA (Steele et al., 1988) over 30 years ago; however, the rapid adoption of dual-energy X-ray absorptiometry (DXA) in the 1990s prevented the commercialization of MRTA at that time (Loucks et al., 2017). Recent reports (Stone et al., 2003 Nov, Wainwright et al., 2005 May) of poor performance of DXA in predicting fractures has led to a decline in confidence in DXA and in insurance coverage for the expense of the procedure. A renewed interest in MRTA has resulted in improvements in the technology, now referred to as Cortical Bone Mechanics Technology (CBMT), which has demonstrated improved accuracy in measurement of bending stiffness when validated against quasistatic mechanical testing (QMT) (Loucks et al., 2017 Aug;, Arnold et al., 2014 Nov 7). This method uses the application of low frequency ( 40–1200 Hz) vibrations at midshaft (Arnold et al., 2014). Additional research is required to demonstrate clinical usefulness of this technology. Currently, there are no FDA-approved devices that measure bone strength or bone stiffness in vivo (Arnold et al., 2014).

## 2 Phase Space Warping

The key hypothesis of this work is that bone fatigue (bone damage accumulation) is a slow-time dynamical process and that such slow-time dynamical processes feedback onto the fast-time dynamics via a quasi-stationary parameter variation (i.e., bone subject to fast-time cyclic loading will accumulate damage and fatigue on a slower time scale) and that the scale separation between fast-time and slow-time dynamics is sufficient such that the fatigue will vary in a quasi-stationary manner with respect to the fast-time dynamics. Thus, the problem of bone fatigue tracking and mitigation reduces to one of extracting slow-time dynamics from fast-time measurements (Chelidze et al., Mar. 2002, Cusumano, Mar. 2002, Chelidze and Liu, Mar. 2005). This work will follow the Phase Space Warping methodology outlined in a series of papers by Chelidze and Cusumano, Sep. 2006, Chelidze and Liu, Mar. 2008.

Formally, it is assumed that the system can be represented as

(1) $$\dot{x}=f\left(x,\mu \left(\phi \right),t\right)$$

(2) $$\dot{\phi }=\epsilon g\left(x,\phi ,t\right)$$

(3) $$y=h\left(x\right)$$

where *x* represents a fast-time observable (bone strain), *ϕ* represents a hidden slow-time variable (bone damage), *μ* represents a system parameter (bone stiffness), *ϵ* represents a small rate constant (measure of scale separation between fast- and slow-time dynamics) and *y* is a scalar measurement taken through the measurement function *h* (strain gage measurement of bone strain).

It is reasonable to assume that *ϵ* is sufficiently smaller than unity and that an intermediate observational time scale may be identified. This time scale is long enough (with respect to the fast-time scale) that sufficient fast-time observations may be obtained to characterize the fast-time system dynamics, but short enough (with respect to the slow-time scale) that *μ* can be assumed constant over the observation interval. The quasi-stationary fast-time dynamics are then characterized through a delay coordinate embedding of the scalar measurements, *y*. Delay coordinate embedding relies on identification of both delay time and embedding dimension (Kantz and Schreiber, 2004). Delay time will be identified through the first minima of average mutual information and embedding dimension will be identified through false nearest neighbor techniques. The delay embedding results in a snap-shot phase space representation of the fast-time dynamics. From this phase space representation, a feature vector is identified which characterizes the fast-time dynamics. For example, each snapshot can be characterized by a particular nonlinear metric, such as characteristic distance (Nguyen et al., 2014), which captures the geometrical features of the attractor and is fast and easy to estimate. This process is repeated for many snap-shots as damage accumulates in the system. The slow-time dynamics are then represented by a slow variation of the feature vector. The feature vectors are concatenated into a tracking matrix and statistical decomposition techniques are applied to identify fatigue modes.

The particular decomposition technique employed is the Smooth Orthogonal Decomposition (SOD), which is a variation on the standard Proper Orthogonal Decomposition (POD) technique (Chelidze and Zhou, May 2006, Kuehl et al., 2014). POD considers the constrained maximization problem

(4) $$\max_{\phi }∥|X\phi ∥|\text{subject to}∥|\phi \phi ∥|=1$$

while SOD considers

(5) $$\max_{\phi }∥|X\phi ∥|\text{subject to}\;\min_{\phi }∥|V\phi ∥|$$

where *X* is the tracking matrix and *V* is the temporal derivative of the tracking matrix. The fundamental difference being that POD identifies statistical modes that optimally account for variance amplitude, whereas SOD identifies modes that optimally account for variance amplitude while at the same time being as smooth in time as possible. SOD has two advantages: 1) Natural phenomena tend to evolve in a smooth manner. 2) SOD modes are sorted based on time scale. Projection of the tracking matrix onto the SOD modes identifies the slow-time damage phase space and the smooth orthogonal coordinates (SOC) reconstruct this phase space. Thus, from fast-time scalar measurements, *y*, one is able to study the slow-time dynamics, *ϕ*. Analysis of the slow-time dynamics is then used to identify the dynamics of damage accumulation and damage mitigation strategies may be employed.

## 3 Experimental methods

**Fig. 1:**
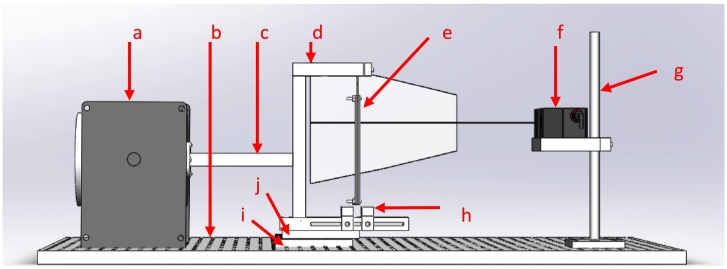
Shaker system testing apparatus: (a) VTS-100 shaker (b) Nexus breadboard (c) 8 in aluminum connector piece (d) aluminum C-frame (e) specimen pendulum (f) LJ-V7300 Keyence laser vibrometer (g) aluminum laser stand (h) Neodymium rare earth magnets or electromagnets (i) aluminum linear guide conversion piece (j) AC-NMS-4 linear guide (Cler, 2016).

The Moon's beam experimental apparatus was designed to apply a quasi-chaotic forcing motion to fatigue specimens (Kuehl and Chelidze, 2008, Kuehl and Chelidze, 2010). This is desirable because bone is not loaded with a constant amplitude and frequency in vivo, and quasi-chaotic forcing motion presents the most difficult manner of fatigue to predict. The testing apparatus is modeled in Fig. 1. A custom aluminum C-frame was mounted to a linear guide to align the motion and eliminate bending moments on the electromagnetic shaker that produced programmable sinusoidal translational motion. The shaker and C-frame were connected with an aluminum bar. A laser vibrometer recorded displacement of the pendulum throughout the fatigue test. The entire system was mounted on a vibration isolation table that isolated it from external vibrations.

The top of the specimen was clamped into the C-frame, and its base was clamped between two rigid steel plates, which created a long magnetic pendulum. The gage length of the test section, thickness of the specimen, and rigidity of the steel plates and top clamp served to prevent higher order modes of oscillation from contaminating the tests. Profile data and direct observation of the test confirmed this. The pendulum hung above two rare-earth permanent magnets, which induced a double potential well on the pendulum. The motion of the pendulum was derived from the combination of the constant amplitude, sinusoidal driving function from the shaker and the action of the magnets on the pendulum and delivered the bending load to the specimen. The magnets provided harmonic, variable, or chaotic motion, depending on the parameters. Here we have chosen to place the system into a state that chaotically mixes ‘large’ bending loads, when oscillating between the magnets, and ‘small’ bending loads, when oscillating over one magnet. The relative amplitudes of the ‘large’ and ‘small’ bending loads are a subtle but important aspect of this study. In previous work (Campbell et al., Dec. 2016, Campbell, 2016), bending loads which correspond to ductile and brittle bone fracture mechanics were identified. Here the systems was tuned such that ‘large’ amplitude bending corresponds to brittle fracture mechanics and ‘small’ amplitude bending corresponds to ductile fracture mechanics. Thus, the experimental setup considered chaotically mixes ductile and brittle fracture mechanics and it is the goal of this work to decouple these fracture mechanisms from bending time series data collected with the laser displacement sensor.

### 3.1 Sample preparation

Human long bones and bovine bone have significantly different properties because human long bones are lamellar bone and bovine long bones are laminar bone (Campbell et al., Dec. 2016, Campbell, 2016). However, because human bone is expensive, requires stringent record keeping, and must be returned to the source at the end of the study, a surrogate material is commonly used in pilot studies. One of the commonly used surrogates is bovine bone, which is relatively inexpensive, has fewer regulatory issues, is easy to acquire, and has a considerable amount of mechanical property data in the literature (Landrigan and Roeder, Jun. 2009, Carter and Hayes, 1977, Behiri and Bonfield, 1989, Abdel-Wahab et al., Jul. 2011).

**Fig. 2:**
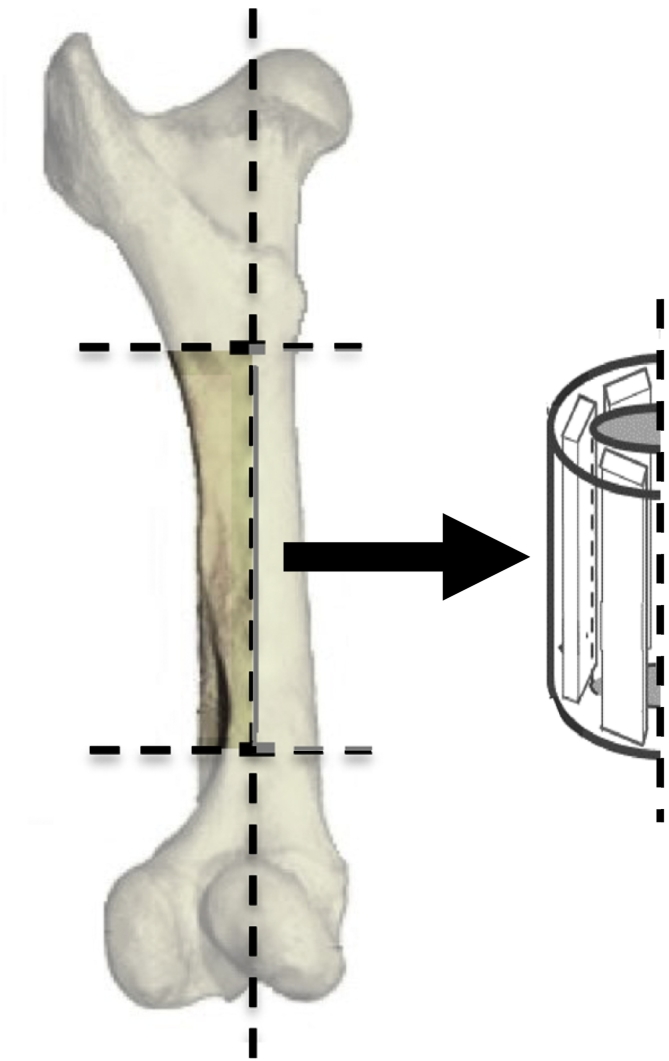
Diagram depicting the location and alignment of the specimens within the bone, where the lateral side of the bone is to the left and the medial side is to the right.

Test specimens were cut from 3 sections of the lateral diaphysis of bovine femurs (see Fig. 2). Each section was cut into 1–2 specimens using a Buehler IsoMetTM 1000 precision saw with a diamond saw blade. Each specimen was approximately 5.08 –7.62 cm ( 2–3 in.) long, 0.25 cm (0.1 in.) thick, and 1.27 cm (0.5 in.) wide. Each specimen was notched with a triangular file across the thickness to reduce the width approximately 3.175 mm (1.25 in.) from the top of the specimen and allowed for the stress to be concentrated in the center of the test section to encourage failure at this point. The specimen width at the notches was 8.128 mm–8.89 mm (0.32 –0.35 in.). A drill press was used to drill a 4.3656 mm (11/64 in.) hole approximately 15.875 mm (0.625 in.) below the notches to accommodate mounting with a nut and bolt of two steel plates that formed the bottom of the pendulum. Seven hours prior to testing, the specimen was set out in the open air to dry. Prior experiments revealed that the resonant frequency of the specimen stabilized after exposure to lab air for 7 h(Campbell et al., Dec. 2016, Campbell, 2016).

### 3.2 Experimental procedure

Once the specimen was mounted on the C-frame, the magnets were adjusted to center the pendulum between them. The pendulum was adjusted in length, and the spacing between the magnets was adjusted to achieve the desired frequency ( 9–10 Hz, within 0.2 Hz of each other) when the pendulum oscillated over either of the magnets (i.e. small amplitude). The frequency of the forcing function driving the shaker was set to the frequency over the potential wells. If the well frequencies were not equal, the average was used. The goal was to set the forcing frequency equal to the resonant frequency over each well because the work done on the system by the applied force would cause the mechanical energy and amplitude of the system's response to increase. The pendulum's swing increased until it was large enough to escape one potential well and fall into the other well. The amplitude of the waveform was slowly increased until chaotic motion was initiated.

## 4 Fracture characterization

**Fig. 3:**
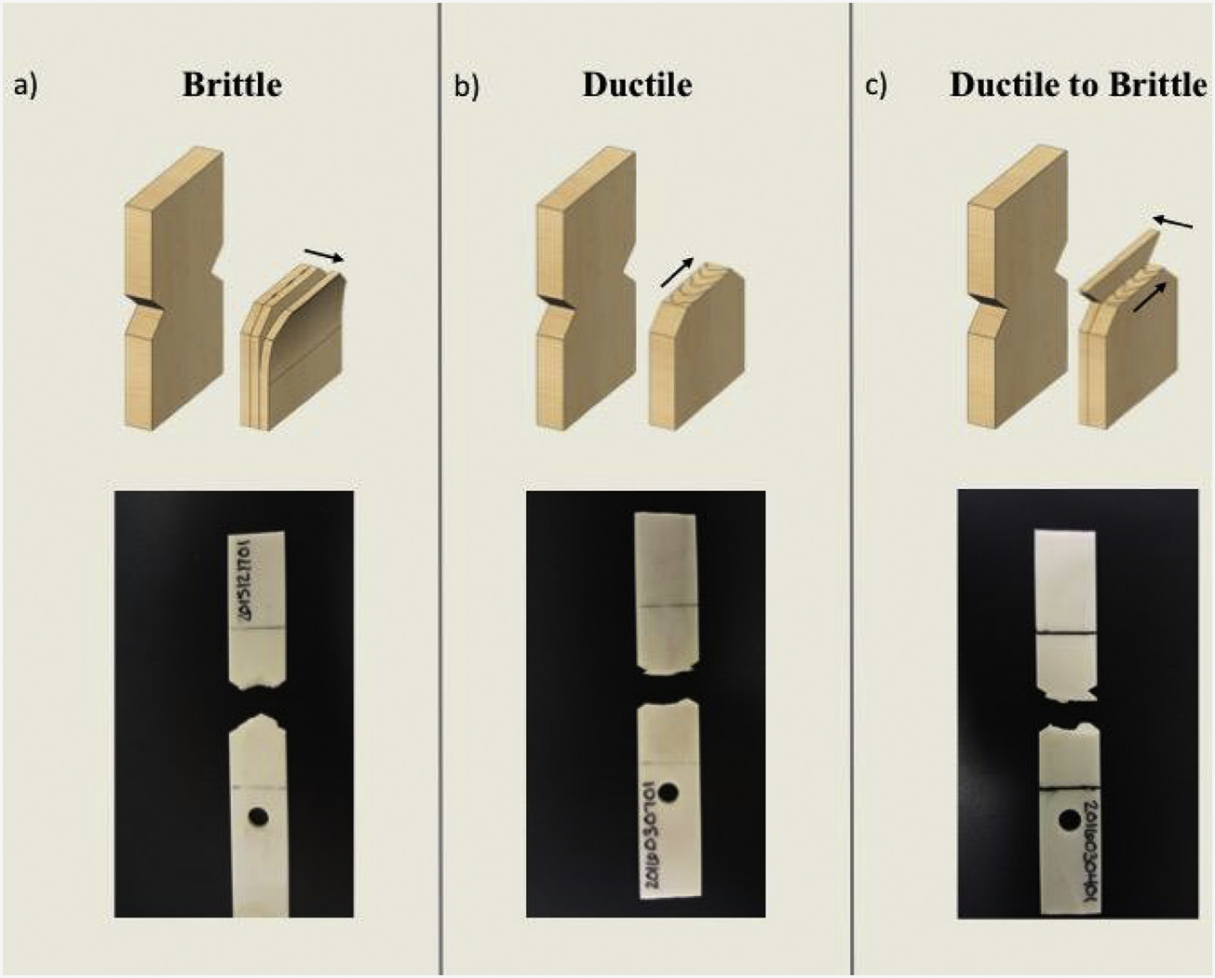
(a) The direction of brittle fracture propagation was through the thickness of the specimen and delamination was apparent. (b) The direction of ductile fracture propagation was across the width of the specimen and beach marking was apparent. (c) Combination fracture surfaces displayed regions that failed by ductile fracture and regions that failed by brittle fracture. Ductile fracture occurred first until the specimen failed by fast brittle fracture.

First, after each specimen failed, it was visually inspected. A photograph of the fracture was taken with a 16-megapixel camera. Based on our knowledge of fracture types from a prior study (Campbell et al., 2016), these images of the fracture surfaces were visually inspected for initial fracture classification (see Fig. 3). Of the 6 specimens considered in this work, one specimen displayed predominantly brittle fracture mechanics, one displayed predominantly ductile fracture mechanics and four displayed a mixture of brittle and ductile mechanics. Brittle breaks are characterized by delaminations which evidence fast fracture mechanics across the specimen's section. Ductile breaks are characterized by beach markings which evidence slow fracture mechanics where crack propagation is apparent. Combination breaks exhibit evidence of both fracture mechanics which manifest as a slow mechanism leading to a fast mechanism. Note, osteon pullout was also observed.

**Fig. 4:**
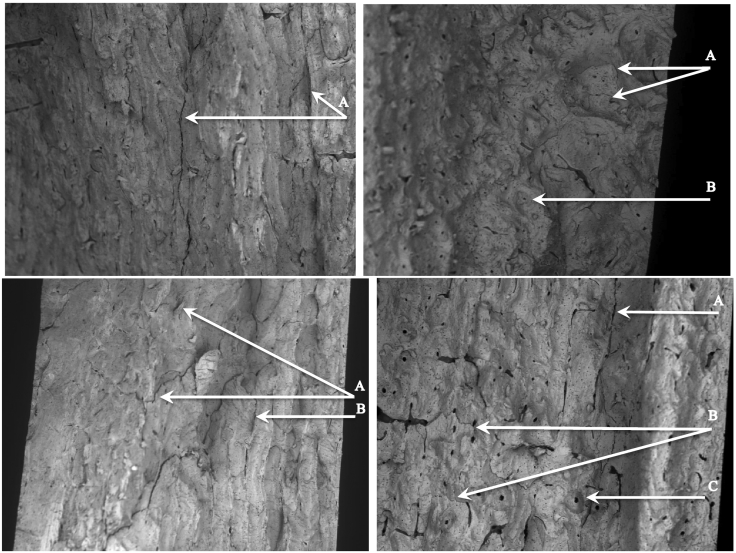
SEM images of different fracture mechanisms. Upper left: A indicates brittle delamination. Upper right: A indicates ductile beach markings and B indicates osteon pullout. Lower left: A indicates beach markings and B indicates delaminations. Lower right: A indicates delamination, B indicates beach markings and C indicates osteon pullout.

Additionally, the fracture surfaces were imaged with a scanning electron microscope, SEM (JEOL, Peabody, MA). The SEM was set to BEC (Bose-Einstein Condensate), spot size 65, and 5 kV. The fracture classification determined previously was confirmed with the SEM images. The brittle breaks displayed delamination between the layers of the bone tissue. The ductile breaks displayed beach markings and osteon pullout. The mixed mode breaks showed a combination of the three. See Fig. 4 for images of brittle, ductile, and combined breaks.

## 5 Results

Visual, SEM and PSW methods were used to analyze the 6 bone fatigue datasets generated in this study. The results of the visual and SEM inspection are summarized in Table 1. As described above, visual and SEM inspection relied on identification of characteristic indicators of relevant fracture mechanisms: beach marking for ductile, delaminations for brittle. Four of the bone specimens displayed a mixture of ductile and brittle fracture mechanics. One specimen displayed purely brittle fracture mechanics and one specimen displayed purely ductile fracture mechanics.

**Table 1:** Visual and SEM based fracture classification of each sample.

Data summary		
Specimen #	Specimen label	Facture type

1	201512701	Brittle
2	2016012101	Ductile/Brittle
3	2016021201	Ductile/Brittle
4	2016021401	Ductile/Brittle
5	2016030401	Ductile/Brittle
6	2016030701	Ductile

**Fig. 5:**
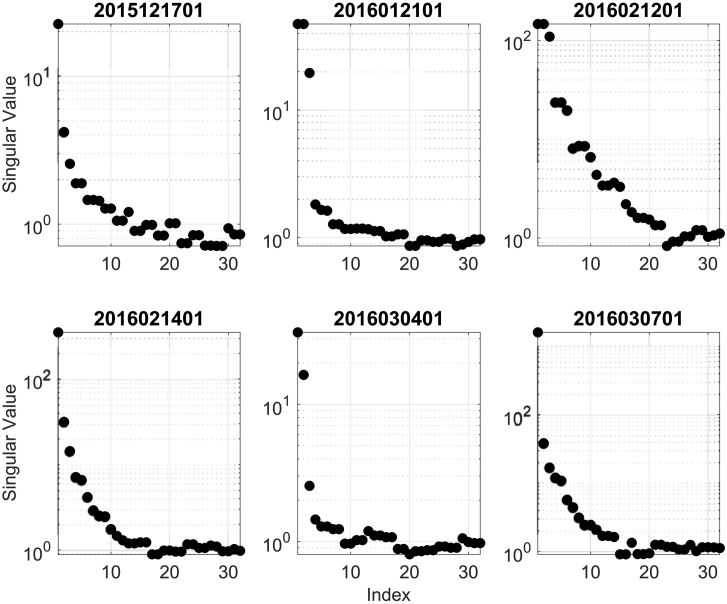
PSW calculated SOVs (eigenvalues) for each specimen.

The output from the PSW analysis takes the form of a SOD decomposition. That is, smooth orthogonal values (SOVs; eigenvalues) and the associated smooth orthogonal coordinates (SOCs; projection of the data onto the smooth orthogonal modes). The SOVs are plotted in Fig. 5. The novelty of SOD analysis is that it partitions the data into modes with common time-scales. The SOVs are related to the inverse square of the mode frequency, when considering pure harmonics. This shows up in the SOVs as modes (or groupings of modes) that operate on a particular time-scale and thus, relevant damage modes can be identified from individual or groups of SOVs that are distinctly isolated from the continuum. Notice samples 2015121701 (Fig. 5 upper left) and 2016030701 (Fig. 5 lower right) each have one dominant SOV, clearly distinct from the continuum. This indicates that one particular mode, and thus one particular damage mechanism, is active. Each of the four other samples possess SOVs (or groupings of SOVs) at two time-scales and thus, it is expected that two damage mechanisms are active. Indeed, this is confirmed by the SEM imaging. It is found that (in this limited dataset) the PSW analysis was able to identify the proper number of damage mechanics active in a chaotically forced (and thus variable amplitude) bone fatigue time-series.

**Fig. 6:**
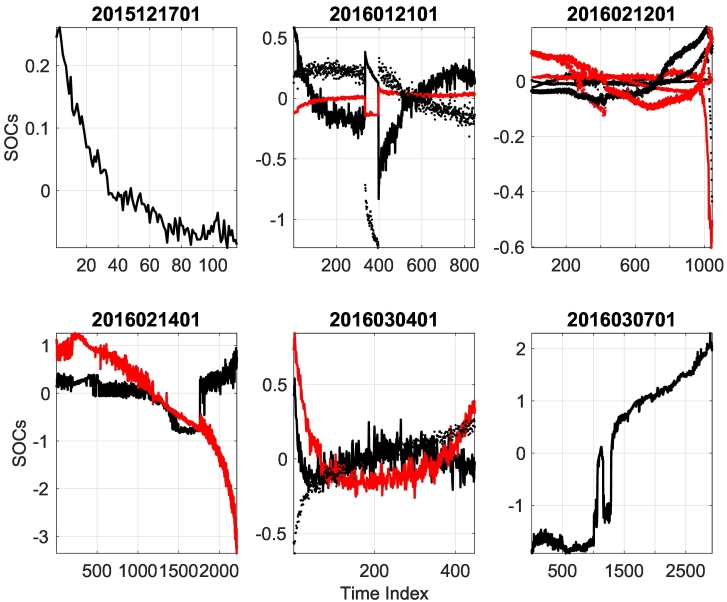
PSW calculated SOCs for each specimen. Lines are colored and textured such that the leading SOV is plotted with a black line, second SOV with a red line, third black dotted, fourth red dotted, fifth black diamond, and sixth red diamond. Only those SOCs with structure distinguishable from noise are plotted. (For interpretation of the references to color in this figure legend, the reader is referred to the web version of this article.)

Along with the distinct time scales identified by the SOVs, additional information is provided from the SOCs. In some cases is it is not particularly clear from the SOVs where the cutoff point between the dynamic modes and the noise floor continuum should be placed. For example, sample 2016030401 might be mistaken as a single dominant fracture mechanism. However, upon inspection of the associated SOCs, it is found that each of the three leading SOVs are valid. This can be seen in Fig. 6 (bottom middle) in that each of the leading three SOCs have distinct trends. Consideration of samples 2015121701 (Fig. 6 upper left) and 2016030701 (Fig. 6 lower right) confirm that only the leading mode contains structure (ie is different than noise).

The SOCs can be thought of as time-series amplitude coefficients of the associated modes. In this limited dataset, we cannot claim to have identified a robust trend in the SOCs data. However, there are several tendencies in the data that should be noted. 1) Brittle fracture mechanics act at high frequencies (*ω*_*B*_), while ductile fracture mechanics operate at lower frequencies (*ω*_*D*_). That is *ω*_*B*_ > *ω*_*D*_. Thus, the corresponding SOVs should obey the relationship SOV_*B*_ < SOV_*D*_. This appears to be confirmed by considering the dominant SOVs from samples 2015121701 (Fig. 5 upper left) and 2016030701 (Fig. 5 lower right). 2) This also indicates that the leading (largest) groupings of SOVs are associated with ductile fracture mechanics while the second groupings are associated with brittle fracture mechanics. 3) There is a tendency in the SOCs evolution (Fig. 6) for the modal amplitude to increase rapidly towards the end of the dataset. It seems reasonable to assume that this rapid increase is associated with the transition from fatigue behavior to material failure/fracture behavior.

## 6 Conclusion

The purpose of this manuscript was to evaluate the effectiveness of PSW for monitoring the health of bone. It was found that PSW is capable of distinguishing different fatigue damage modes. The graphs of the SOVs, equivalent to the inverse of the frequency squared, reveal the different damage modes and their time scales. Relevant damage modes can be identified from individual or groups of SOVs that are distinctly isolated from the continuum. The frequencies that are revealed by these groupings agree with the damage types identified with the SEM images for each test. Larger eigenvalues correspond to a lower-frequency damage accumulation while the smaller eigenvalues correspond to a higher-frequency of damage accumulation. The high-frequency/fast-time scale for damage acquisition corresponds to a brittle damage mode, and the low-frequency/slow-time scale for damage acquisition corresponds to a ductile damage mode. The division between SOV amplitudes that correspond to brittle or ductile fractures in any given experiment is clear, but further research is needed to develop robust statistics for inter-experimental comparison. Though not established with statistical significance in this study, the tendency for SOCs amplitude to rapidly increase before material failure was observed.

To the author's knowledge, the method of PSW had never been applied to bone tissue. The research conducted here indicates that the PSW methodology shows promise for use as a tool to monitor bone health and to predict the fatigue life of bone. The researchers feel this could be a valuable tool for monitoring bone health of total joint replacement patients, the elderly who are at high-risk for bone fracture and astronauts during extended spaceflight missions.

## Transparency document

Transparency document. Image 1

## Declaration of Competing Interest

The authors are aware of no conflicts of interest related to the publication of the manuscript “A novel method for bone fatigue monitoring and prediction” in Bone Reports.
